# osFP: a web server for predicting the oligomeric states of fluorescent proteins

**DOI:** 10.1186/s13321-016-0185-8

**Published:** 2016-12-20

**Authors:** Saw Simeon, Watshara Shoombuatong, Nuttapat Anuwongcharoen, Likit Preeyanon, Virapong Prachayasittikul, Jarl E. S. Wikberg, Chanin Nantasenamat

**Affiliations:** 1 Center of Data Mining and Biomedical Informatics, Faculty of Medical Technology, Mahidol University, Bangkok, 10700 Thailand; 2 Department of Community Medical Technology, Faculty of Medical Technology, Mahidol University, Bangkok, 10700 Thailand; 3 Department of Pharmaceutical Biosciences, Uppsala University, 751 24 Uppsala, Sweden

**Keywords:** Fluorescent protein, FP, Green fluorescent protein, GFP, Oligomeric state, Data mining, Web server

## Abstract

**Background:**

Currently, monomeric fluorescent proteins (FP) are ideal markers for protein tagging. The prediction of oligomeric states is helpful for enhancing live biomedical imaging. Computational prediction of FP oligomeric states can accelerate the effort of protein engineering efforts of creating monomeric FPs. To the best of our knowledge, this study represents the first computational model for predicting and analyzing FP oligomerization directly from the amino acid sequence.

**Results:**

After data curation, an exhaustive data set consisting of 397 non-redundant FP oligomeric states was compiled from the literature. Results from benchmarking of the protein descriptors revealed that the model built with amino acid composition descriptors was the top performing model with accuracy, sensitivity and specificity in excess of 80% and MCC greater than 0.6 for all three data subsets (e.g. training, tenfold cross-validation and external sets). The model provided insights on the important residues governing the oligomerization of FP. To maximize the benefit of the generated predictive model, it was implemented as a web server under the R programming environment.

**Conclusion:**

osFP affords a user-friendly interface that can be used to predict the oligomeric state of FP using the protein sequence. The advantage of osFP is that it is platform-independent meaning that it can be accessed via a web browser on any operating system and device. osFP is freely accessible at http://codes.bio/osfp/ while the source code and data set is provided on GitHub at https://github.com/chaninn/osFP/.

**Graphical Abstract:**
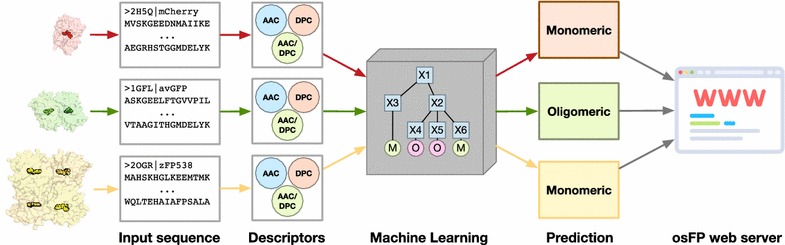
.

## Background

Many coral fluorescent proteins (FP) are observed in anthozoans and because of the fact that their tertiary structures are homologous to the *Aequorea victoria* jellyfish, they are often referred to as green fluorescent protein (GFP)-like. These FPs represent an important class of bioluminescent proteins because of their immense utility for biomedical imaging in the life sciences. Such popularity lies in the diversity of their spectral colors and their independence from co-factors owing to the autocatalytic post-translational modifications of the three or four amino acid precursors of the chromophore. Although the inherently weak dimerization of *Aequorea* GFP does not hinder its usage as a protein tag, the obligate tetrameric structure of DsRed has greatly impeded its utilization as a genetically encoded fusion tag because of possible perturbations to the tagged protein. Although oligomeric FPs in corals can serve as “sunscreen” to prevent coral bleaching, steric conflicts and stoichiometric clashes can occur when DsRed is tagged to oligomeric protein of interest (e.g. actin, tubulin, connexin or histone) [1].

Despite being the essential tagging tool for live biomedical imaging, the oligomerization of FPs hinders their utilization, problems have been reported, such as abnormal localizations, perturbing normal functions, interfering with signaling cascades, and preventing normal oligomerization fusion products within specific organelles. Shcherbo et al. [2] stressed that Katushaka, the dimeric far-red mutants of FPs from the sea anemone *Entacmaea quadricolor*, can form abnormal localization in Phoenix eco cells. Mizuno et al. [3] demonstrated that aggregation of DsRed disturbs normal function of calmodulin in the cytosol. Zacharias [4] stressed that oligomerization of FPs interfered with target protein signaling cascades when using them as tagging probes for fluorescence resonance energy transfer (FRET). Lauf et al. [5] showed that tetrameric DsRed tagged with connexins led to problems because DsRed crosslinked between different connexins, negatively affected the connexin function. Jain [6] reported that in the secretory pathway of endocrine cells, EGFP oligomerized through the disulphide-linkage of Cys49 and Cys71. Typically, there are two ways to overcome oligomerization problems: modify the FPs to monomeric states through rational and/or random mutagenesis or look for natural monomeric FPs from other organisms. In regards to the former, Zacharias [4] converted the weak dimeric *A. victoria* FP to the monomeric form by scrutinizing the crystal structure of GFP and replacing hydrophobic residues with polar charged amino acids. As for the latter approach, Shagin et al. [7] screened for FPs from hydrozoan species from the ocean and observed that one in six copepoda FPs were monomeric.

Quantitative structure-property relationship (QSPR) represents an important paradigm that allows the prediction of biological and chemical properties of interests as a function of their physicochemical properties through the use of machine learning methods [8–10]. Garian [11] proposed one of the first study for predicting protein oligomerization using decision tree (DT) in which primary sequences of proteins from the SWISS-PROT database (Release 34) were classified as homodimers or non-homodimers. Afterwards, several computational models based on different machine learning algorithms were then reported such as support vector machines (SVM) [12–14], function of degree of disagreement (FDOD) [15], *k*-NN algorithm [16] and probability approaches [17]. Details of existing methods for predicting protein oligomerization properties are provided in Table 1. Although several predictive models have been reported for predicting protein oligomerization, however no computational studies exists for specifically analyzing and investigating FPs.

**Table 1:** Summary of existing studies for predicting oligomeric states from protein sequences

Data set	Method	Internal set size	External set size	Sequence features	Source
SWISS-PROT (release 34)	DT	1639	N/A	PCP	[11]
	SVM	1639	N/A	AAC, AC	[14]
	FDOD	1639	N/A	QSO	[15]
SWISS-PROT (release 34) after removing similar protein sequence	SVM	1568	N/A	QSO	[13]
	SVM	1568	N/A	AAC, DPC, AACD	[21]
	k-NN	1568	N/A	QSO	[16]
	SVM	1568	1283	PseAAC	[12]
SWISS-PROT (release 40)	DA	3174	332	PseAAC	[22]
	SVM	3174	N/A	FS, MSE	[23]
	NN	3174	332	PseAAC	[24]
UniProtKB (release 15.6)	Probability	5495	N/A	AAC, DPC	[17]
	Fuzzy *k*-NN	5495	N/A	PseAAC	[25]
SWISS-PROT (release 55.3)	OET-*k*-NN	6702	N/A	FunD, PsePSSM	[26]
	$\hbox{DWT}\_\hbox{DT}$	6702	N/A	PseAAC, PCP	[27]
FP data set	DT	318	79	AAC, DPC, TPC	This study
				AC, CTD, Ctriad	
				QSO, PseAAC	

*DT* decision tree, $\textit{DWT}\_{ DT}$ discrete wavelength transform and decision tree, *FDOD* function of degree of disagreement, *DA* discriminatory analysis, *SVM* support vector machine, *NN* neural network, *k-NN k*-nearest neighbors, *Fuzzy k-NN* Fuzzy *k*-nearest neighbors, *OET*-*k-NN* optimized evidence-theoretic *k*-NN algorithm, *AAC* amino acid composition, *AACD* amino acid composition distribution, *AC* autocorrelation descriptors derived from several physicochemical properties including Geary, Moreau-Broto and Moran, *APseAAC* amphiphilic pseudo-amino acid composition, *CTD* composition, transition and distribution, *Ctriad* conjoint triad descriptors, *DPC* dipeptide composition, $\textit{DWT}\_{ DT}$ discrete wavelet transform and decision tree, *FDOD* function of degree of disagreement, *FS* the factor scores, *FunD* functional domain composition, *MSE* multi-scale energy, *PCP* physicochemical properties, *PseAAC* pseudo amino acid composition, *PsePSSM* pseudo position-specific score matrix, *TPC* tripeptide composition, *QSO* quasi-sequence-order descriptors

To the best of our knowledge, this study proposes the first computational model for predicting the oligomeric states directly from the protein sequence of a large set of FPs compiled from the literature. It is also worthy to note that the sample size of this study is comparatively smaller than the aforementioned studies on protein oligomerization but such disparity is limited by the currently available experimental data on FP oligomerization. Current machine learning methods that are being used to construct predictive models for predicting the protein oligomeric state ranges from simple and interpretable approach (e.g. DT) to more sophisticated (e.g. NN, SVM, etc.) approaches. As this study aims for simple and interpretable predictive models, the DT approach was employed for classifying FPs as being either monomeric or oligomeric. In spite of its simplicity, most of the predictive models built as a function of various classes of protein descriptors afforded robust performance as deduced by the statistical parameters. The best model was further developed as the osFP web server that is freely available at http://codes.bio/osfp/. As to encourage further developments and extension of the predictive model and web server, the source code, complete data set and example files are provided on the repository page of osFP on GitHub at https://github.com/chaninn/osFP/.

## Methods

### Data sets

A data set consisting of 409 FPs along with their oligomeric states were compiled from the primary literature and is available on the osFP repository page on GitHub at https://github.com/chaninn/osFP/. Monomeric FPs are ideal tools for fluorescent tagging in biomedical imaging whereas oligomeric FPs hinder their usage as tagging labels because of their tendencies to aggregate. Therefore, we aimed to classify the FPs as being either in the monomeric or oligomeric states.

Redundant sequences in the training or testing data may lead to overestimation of the model in which the learning method could only reproduce its own input data rather than being able to interpolate and extrapolate [18]. Without considering the homology relatedness, the predictive performance will be inflated. In fact, many of the FPs were obtained via site-directed mutagenesis from just a few wild-type sequences. For example, the Ala206Lys mutation in green fluorescent protein from *Aequorea victoria* caused the FP to change from oligomeric (weak dimer) to the monomeric state. Thus, it is important to consider homology reduction for the sequence-based classification performed herein. Redundancy reduction of the sequence was performed using the CD–HIT algorithm [19] as implemented in the *cdhitHR* function of the *BioSeqClass* R package [20]. Threshold values of 0.95, 0.99 and 1.00 (i.e. corresponding to 95%, 99% and 100%, respectively) was set using the *identity* argument to produce a reduced subset consisting of 136, 261 and 397 FPs, respectively.

The data set was randomly divided into two subsets consisting of an internal set (80%) and an external set (20%) in which the former set was used to constructing predictive models as full training and tenfold cross-validation (tenfold CV) while samples in the latter set was predicted using the aforementioned trained model. This data splitting was performed for 100 iterations followed by computing the mean values for the performance metrics.

### Protein descriptor calculation

Several classes of amino acid descriptors consisting of 8420 amino acid/dipeptide/tripeptide composition (AAC/DPC/TPC) descriptors (i.e. 20 amino acid composition, 400 dipeptide composition and 8000 tripeptide composition descriptors, 720 autocorrelation (AC) descriptors (i.e. 240 normalized Moreau-Broto autocorrelation, 240 Moran autocorrelation and 240 Geary autocorrelation descriptors), 147 composition, transition and distribution (CTD) descriptors, 343 conjoint triad (Ctriad) descriptors, 160 quasi-sequence-order (QSO) (i.e. 60 sequence-order-coupling number and 100 quasi-sequence-order descriptors) and 130 pseudo-amino acid composition (PseAAC) descriptors (i.e. 50 pseudo-amino acid composition and 80 amphiphilic pseudo-amino acid composition) were used to represent features of the amino acid sequence.

The amino acid/peptide composition descriptors were computed using *extractAAC*, *extractDC* and *extractTC* functions from the *protr* R package. Amino acid composition is the proportion of all twenty naturally occurring amino acids, dipeptide composition constitutes 400 possible sequence of dipeptides and tripeptide composition encompasses 8000 possible sequence of tripeptides. These three sets of descriptors can be defined by the following equations:

1 $$\begin{aligned} f(r) = \frac{N_r}{N} \quad r = 1, 2, \ldots , 20. \end{aligned}$$

where $N_r$ is the number of amino acid type *r* and *N* is the length of the sequence.

2 $$\begin{aligned} f(r, s) = \frac{N_{rs}}{N - 1} \quad r, s = 1, 2, \ldots , 20. \end{aligned}$$

where $N_{rs}$ is the number of dipeptides represented by amino acid types *r* and *s*.

3 $$\begin{aligned} f(r, s, t) = \frac{N_{rst}}{N - 2} \quad r, s, t = 1, 2, \ldots , 20 \end{aligned}$$

where $N_{rst}$ is the number of tripeptides represented by amino acid types *r*, *s* and *t*.

AC descriptors were computed using *extractMoreauBroto*, *extractMoran* and *extractGeary* functions from the *protr* R package [28] to obtain the normalized Moreau-Broto autocorrelation, Moran autocorrelation and Geary autocorrelation descriptors, respectively. The AC descriptors are defined based on the distribution of the physicochemical properties of amino acid, which can be derived from the AAindex database. The normalized Moreau-Broto autocorrelation is defined by 8 physicochemical properties consisting of normalized average hydrophobicity scales, average flexibility indices, polarizability parameter, free energy of solution in water, residue accessible surface area in tripeptide, residue volume, steric parameter, relative mutability with respective AAindex database ID of CIDH920105, BHAR880101, CHAM820101, CHAM820102, CHOC760101, BIGC670101, CHAM810101 and DAYM780201, respectively.

Moreau-Broto autocorrelation descriptor is summarized below:

4 $$\begin{aligned} AC(d) = \sum _{i=1}^{N-d} P_i P_{i + d} \quad d = 1, 2, \ldots , 30 \end{aligned}$$

where *d* is the lag of the autocorrelation while $P_i$ and $P_{i+d}$ are properties of the amino acids at positions *i* and $i+d$, respectively.

Moran autocorrelation descriptors can be defined as follows:

5 $$\begin{aligned} I(d) = \frac{\frac{1}{N-d} \sum _{i=1}^{N-d} (P_i - \bar{P}') (P_{i+d} - \bar{P}')}{\frac{1}{N} \sum _{i=1}^{N} (P_i - \bar{P}')^2} \quad d = 1, 2, \ldots , 30 \end{aligned}$$

where *d*, $P_i$ and $P_{i+d}$ are as defined above while $\bar{P}'$ is the considered property *P* along the sequence:

6 $$\begin{aligned} \bar{P}' = \frac{\sum _{i=1}^N P_i}{N} \end{aligned}$$

where *d*, *P*, $P_i$ and $P_{i+d}$ are as described above.

Geary autocorrelation descriptors are defined as follows:

7 $$\begin{aligned} C(d) = \frac{\frac{1}{2(N-d)} \sum _{i=1}^{N-d} (P_i - P_{i+d})^2}{\frac{1}{N-1} \sum _{i=1}^{N} (P_i - \bar{P}')^2} \quad d = 1, 2, \ldots , 30 \end{aligned}$$

where *d*, *P*, $P_i$ and $P_{i+d}$ are as mentioned previously.

CTD descriptors were computed using *extractCTDC*, *extractCTDT* and *extractCTDD* functions from the *protr* R package to produce the composition, transition and distribution descriptors, respectively. Briefly, the amino acid were categorized according to their properties (i.e. hydrophobicity, normalized van dar Waals volume, polarity, polarizability, charge, secondary structure and solvent accessibility) which are denoted as sub-class 1, sub-class 2 and sub-class 3. The composition descriptors is the global percentage for each class of each sequence. Depending on the sub-class, the sequence were encoded using the following equation:

8 $$\begin{aligned} C_r = \frac{n_r}{n} \quad r = 1, 2, 3 \end{aligned}$$

where $n_r$ is the number of amino acid type *r* in the encoded sequence and *N* is the length of the sequence. Transition is the percent frequency of a transition from one category to another, which can be calculated as follows:

9 $$\begin{aligned} T_{rs} = \frac{n_{rs} + n_{sr}}{N - 1} \quad rs = \text {`12'}, \text {`13'}, \text {`23'} \end{aligned}$$

where $n_{rs}$ and $n_{sr}$ are the numbers of dipeptide encoded as “rs” and “sr”, respectively, in the sequence and *N* is the length of the sequence. Distribution descriptor describes the chain length in which the first residue as well as 25, 50, 75 and 100% of amino acids reside for a specified encoded class.

Ctriad descriptors were obtained using the *extractCTriad* function from the *protr* R package. The conjoint triad descriptors are abstracts descriptors of protein pairs based on the categories of amino acid. The twenty natural amino acids were catogerizied based on their dipoles and volumes of the side chains because electrostatic and hydrophobic interactions, respectively, play an important part in protein–protein interaction. These two parameters were calculated via density-functional theory method B3LYP/6-31G and molecular modeling approach. The amino acids are then further categorized into seven classes based on their dipoles and values of their respective side chains. Triads can be defined as a unit of any three continuous amino acids, considering the properties of sandwiched amino acid and its vicinal amino acids.

QSO descriptors were computed using *extractSOCN* and *extractQSO* functions from the *protr* R package. The Quasi-Sequence-Order descriptors based from the distance matrix between twenty amino acids as proposed by [29].

PseAAC descriptors were calculated using *extractPAAC* and *extractAPAAC* functions of the *protr* R package. PseAAC can also be called type 1 pseudo-amino acid composition as they are based on the original hydrophobicity values, hydrophilicity and side chain masses, which can be summarized as follows:

10 $$\begin{aligned} H_1 (i) = \frac{H_1^o (i) - \frac{1}{20} \sum _{i=1}^{20} H_1^o (i)}{\sqrt{\frac{\sum _{i=1}^{20} [H_1^o (i) - \frac{1}{20} \sum _{i=1}^{20} H_1^o (i) ]^2}{20}}} \end{aligned}$$

where $H_1^o (i)$, $H_2^o (i)$ and $M^o (i)$ ($i=1, 2, 3, \ldots , 20$) represents the hydrophobicity values, the hydrophilicity values and the original side chain masses of the 20 naturally occurring amino acids.

The APseAAC, also known as type 2 pseudo-amino acid composition, is defined by the following equation:

11 $$\begin{aligned} \begin{aligned} H_{i, j}^1&= H_1 (i) H_1 (j)\\ H_{i, j}^2&= H_2 (i) H_2 (j) \end{aligned} \end{aligned}$$

where $H_1 (i)$ and $H_2 (j)$ represents hydrophobicity and hydrophilicity, respectively.

From these qualities, sequence order factors can be defined as follows:

12 $$\begin{aligned} \begin{aligned} \tau _1&= \frac{1}{N-1} \sum _{i=1}^{N-1} H_{i, i+1}^1\\ \tau _2&= \frac{1}{N-1} \sum _{i=1}^{N-1} H_{i, i+1}^2\\ \tau _3&= \frac{1}{N-2} \sum _{i=1}^{N-2} H_{i, i+2}^1\\ \tau _4&= \frac{1}{N-2} \sum _{i=1}^{N-2} H_{i, i+2}^2\\&\ldots \\ \tau _{2 \lambda - 1}&= \frac{1}{N-\lambda } \sum _{i=1}^{N-\lambda } H_{i, i+\lambda }^1\\ \tau _{2 \lambda }&= \frac{1}{N-\lambda } \sum _{i=1}^{N-\lambda } H_{i, i+\lambda }^2 \end{aligned} \end{aligned}$$

A set of APseAAC descriptors can be defined as:

13 $$\begin{aligned} P_c & = \frac{f_c}{\sum _{r=1}^{20} f_r + w \sum _{j=1}^{2 \lambda } \tau _j} \quad (1< c < 20) \end{aligned}$$

14 $$\begin{aligned} P_c & = {} \frac{w \tau _u}{\sum _{r=1}^{20} f_r + w \sum _{j=1}^{2 \lambda } \tau _j} \quad (21< u < 20 + 2 \lambda ) \end{aligned}$$

where *w* is the weighting factor and is taken as $w = 0.5$.

Thus, six descriptor classes consisting of AAC/DPC/TPC, AC, CTD, Ctriad, QSO and PseAAC were benchmark for its ability to predict the oligomeric states of FP.

### Feature selection

Intercorrelation (or collinearity) is a condition where pairs of descriptors have a major correlation with each others. It has negative impact on the analysis as highly correlated predictors add more complexity to the model than information they provide. In addition, one of the key principle in the analysis of high dimensional data, which is also known as the *curse of dimensionality* that tempts practitioners to fall into a trap in which the inclusion of a higher number of features will yield higher performance for the predictive model. Indeed, adding additional features that are truly associated with the outcome (e.g. oligomerization) is expected to improve the predictive model. On the other hand, the addition of noise features that are not truly relevant to the outcome is expected to deteriorate the model thereby leading to a reduction of the model performance. This is because the incorporation of noise features tends to increase the risk of overfitting. As low collinearity is favorable for retaining a non-redundant set of descriptors and as there is no strict criteria on the removal threshold, therefore typically high threshold value for the correlation coefficient are employed. Cronin and Schultz et al. [30] pointed out that there seems to be no consensus on the threshold criterion for the correlation coefficient as acceptable values ranged from less than 0.4 to 0.9. Thus, the *cor* function from the *caret* R package [31] was used to calculate correlations between descriptors. Subsequently, collinear descriptors were removed using an arbitrary threshold of 0.7 for the Pearson’s correlation coefficient as implemented by the *findCorrelation* function from the *caret* R package. Such threshold value is deemed to be a stringent value for exclusion of descriptors displaying mild intercorrelation with one another whereas a high threshold value of 0.9, for instance, would allow fewer removal of descriptors while allowing descriptor pairs with mild intercorrelation to be included in the model.

### Multivariate analysis

A decision tree (DT) algorithm was utilized for constructing a computational model to predict FP oligomeric states. Because the DT method affords interpretable rules for estimating feature importance pertaining to FP oligomeric states, it is helpful in revealing the different characteristics between monomeric and oligomeric states. The construction of a DT model requires the following: (i) all samples in the internal set belong to a single class; (ii) the tree depth is close to maximum; and (iii) the number of classes in the terminal node is less than the minimum number of classes of the parent nodes. In general, the root node is a variable with the highest information gain, whereas the other internal nodes provide the second and subsequent highest information gain thereafter. Machine learning models were built in the R statistical programming language using the *J48* function from the *RWeka* R package.

### Statistical assessment of predictive model

For any empirical learning method, statistical assessment of the model robustness is an important process. Four measurements were used to evaluate the prediction performances of the proposed model: accuracy (Ac), sensitivity (Sn), specificity (Sp) and Matthews’ correlation coefficient (MCC). These parameters are defined as follows:

15 $$\begin{aligned} Ac & = {} \frac{TP + TN}{(TP + TN + FP + FN)}\times 100 \end{aligned}$$

16 $$\begin{aligned} Sn & = {} \frac{TP}{(TP+ FN)}\times 100 \end{aligned}$$

17 $$\begin{aligned} Sp & = {} \frac{TN}{(TN+ FP)}\times 100 \end{aligned}$$

18 $$\begin{aligned} MCC & = {} \frac{TP\times TN - FP\times FN}{\sqrt{(TP+FP)(TP+FN)(TN+FP)(TN+FN)}} \end{aligned}$$

where TP is the instances of true positives, TN is the instances of true negatives, FP is the instances of false positives and FN is the instance of false negatives. In this study, a tenfold CV procedure was used to confirm the reliability and robustness of the QSPR models using the training set. Additionally, external validation set was used to assess the generalizability to our proposed model for predicting unknown samples. It should be noted that the range of MCC is from –1 to 1 in which a value of 1 indicates the best possible prediction while –1 indicates the worst possible prediction. On the other hand, a value of 0 suggests the occurrence of random prediction.

### Development of the osFP webserver

The osFP web server was developed using the web application framework known as *Shiny* under the R statistical programming language. Technically, the Shiny web application framework is comprised of two components: (i) ui.R (i.e. the user interface script) and (ii) server.R (i.e. the server script). The user interface script is responsible for producing the layout of the web application that users can see and interact (i.e. entering the input sequence of FP in FASTA format for calculation submission) while the server script performs the calculations and generates the output (i.e. prediction results of the oligomeric state). As it is computationally intensive to compute 8420 descriptors (i.e. 20 amino acid, 400 dipeptide and 8000 tripeptide composition descriptors), only the top 20 important features as revealed by the DT model were used in the production environment (i.e. the osFP web server). As such, this required slight adaptation to the descriptor calculation functions from the *protr* R package, particularly by computing only specific descriptors from the list of the top 20 important features instead of the default total number of 8420 descriptors for the three descriptor classes.

osFP is hosted on a Ubuntu Linux server via the the cloud infrastructure provider, DigitalOcean. The benefits of hosting on the cloud is many: (i) low start-up cost (i.e. no need for costly investments on hardware, no maintenance cost and no need for server administrator), (ii) scalable resources (i.e. when the need for more RAM or storage arises the server can be upgraded) (iii) operating systems are pre-installed and available in several Linux distributions (i.e. no need for lengthy installation of the operating system as a working server takes under a minute to be provisioned), (iv) full access and control of the server (i.e. freedom to install and configure softwares) and (v) the whole server can be backed up as an image.

The provisioned web server used to host osFP is based on Ubuntu version 14.10. Firstly, the R base software and associated packages (i.e. shiny, shinythemes, shinyjs, protr, seqinr, RWeka and markdown, which are used on the osFP web server) were installed via the apt-get package handling utility in the command line. Secondly, the RStudio Shiny Server, which is available at https://www.rstudio.com/products/shiny/download-server/, was installed. At default, the directory for housing Shiny applications is set to /srv/shiny-server/ while the Shiny application would typically run at port number 3838, therefore the base URL will look something like http://192.168.1.1:3838/ where 192.168.1.1 represents the IP address while the full URL would look something like http://192.168.1.1:3838/osfp/. There is a workaround to hiding the port number but one needs to configure the Shiny configuration file (i.e. available at /etc/shiny-server/shiny-server.conf) and/or the Apache server settings (i.e. available at /etc/apache2/sites-available/). There is an excellent step-by-step tutorial provided by the DigitalOcean user community on installing and configuring the Shiny server [32]. Although, the Shiny server supports the use of databases such as MySQL, however the simplicity and moderate size of the data set employed herein makes satisfactory use of the CSV file format to store, retrieve and analyze the data via functionalities of the R environment.

## Results and discussion

### Predicting FP oligomeric states

**Fig. 1:**
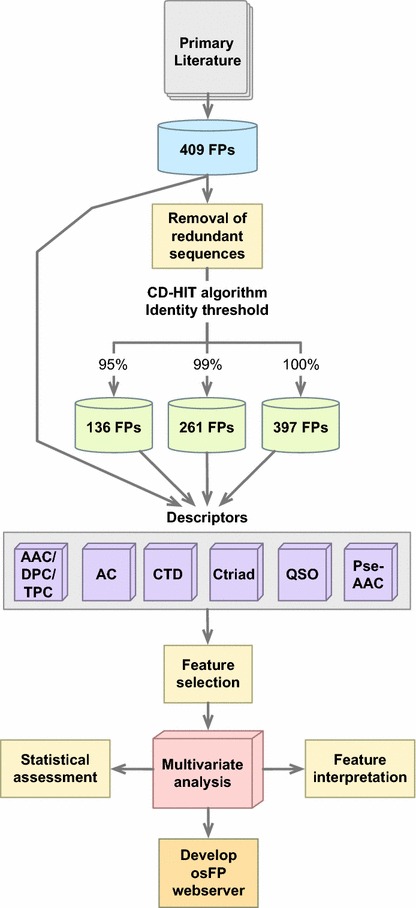
Workflow of QSPR modeling for predicting oligomeric states of FP

Figure 1 illustrates the flowchart of the workflow used to predict and analyze the oligomerization of FPs. In the study, six classes of protein features were benchmark to provide a better picture on which protein features can be considered sufficient to provide insights on the oligomerization of FPs. To avoid the possibility of obtaining prediction results that may arise from chance correlation from a single calculation, the multivariate analysis was performed for 100 independent iterations where each run involves random data splitting to an internal and external sets consisting of 80 and 20%, respectively.

Judging from the performances, although different descriptor sets may capture different aspects of amino acids however all descriptor sets afforded similar level of performance. This may indicate that all descriptor sets capture the oligomerization space well and can be used as a features to train predictive QSPR models as assessed via Ac, Sn, Sp and MCC. However, the best performing method was AAC/DPC/TPC descriptors whereas AC descriptors ranked last, indicating that the amino acid composition descriptors are capable of capturing information on the oligomerization of FP. The predictive performance of the six classes of protein descriptors were further discussed in the following paragraphs.

The internal set was used to construct a predictive model by means of the J48 algorithm as to discriminate FPs to either monomers or oligomers. The predictive model was fine tuned using tenfold CV as to prevent overtraining on the internal set and then tested on an external set in order to assess its ability to accurately predict unknown samples. Table 2 provides the mean performance comparison amongst the various types of protein descriptors as assessed by the training set, the tenfold CV set and the external set.

**Table 2:** Summary of performance of QSAR models for predicting the oligomeric state of FPs (100% homologous sequence reduction) using the J48 algorithm

Descriptors	Training set				Tenfold CV set				External set			
	Ac (%)	Sn (%)	Sp (%)	MCC	Ac (%)	Sn (%)	Sp (%)	MCC	Ac (%)	Sn (%)	Sp (%)	MCC
AAC/DPC/TPC	97.40 ± 0.77	97.72 ± 1.23	97.14 ± 1.31	0.95 ± 0.02	83.07 ± 2.04	83.26 ± 2.19	82.95 ± 2.27	0.66 ± 0.04	83.26 ± 3.58	83.77 ± 5.14	83.37 ± 4.45	0.67 ± 0.07
AC	98.70 ± 0.58	98.54 ± 0.93	98.86 ± 0.75	0.97 ± 0.01	78.36 ± 2.35	78.36 ± 2.36	78.67 ± 2.30	0.57 ± 0.04	78.49 ± 4.76	78.65 ± 5.66	78.89 ± 5.62	0.57 ± 0.10
CTD	97.58 ± 0.82	98.05 ± 0.99	97.20 ± 1.33	0.95 ± 0.02	80.28 ± 2.37	79.66 ± 2.98	80.88 ± 2.33	0.60 ± 0.05	80.40 ± 4.92	80.37 ± 6.63	81.01 ± 5.34	0.61 ± 0.10
Ctriad	95.46 ± 1.08	96.20 ± 1.51	94.85 ± 1.87	0.91 ± 0.02	80.38 ± 2.01	81.07 ± 2.43	79.82 ± 2.08	0.61 ± 0.04	81.06 ± 4.83	81.80 ± 5.79	80.98 ± 5.55	0.62 ± 0.10
QSO	98.35 ± 0.63	98.35 ± 0.83	98.36 ± 0.93	0.97 ± 0.01	80.15 ± 1.91	80.11 ± 2.25	80.27 ± 2.16	0.60 ± 0.04	81.42 ± 4.00	81.86 ± 5.21	81.58 ± 5.00	0.63 ± 0.08
PseAAC	98.51 ± 0.62	98.63 ± 0.85	98.42 ± 1.05	0.97 ± 0.01	81.13 ± 1.89	81.19 ± 2.24	81.14 ± 2.09	0.62 ± 0.04	81.40 ± 4.66	81.13 ± 5.48	82.19 ± 5.54	0.63 ± 0.09

It was observed that models built with AAC/DPC/TPC outperformed the others with Ac, Sn, Sp and MCC of 83.01 ± 2.04%, 83.26 ± 2.19%, 82.95 ± 2.27% and 0.66 ± 0.04, respectively, for the tenfold CV set. Furthermore, AAC/DPC/TPC also afforded the best performance on the external set with Ac, Sn, Sp and MCC of 83.26 ± 3.58%, 83.77 ± 5.14%, 83.37 ± 4.45% and 0.67 ± 0.07, respectively. On the other hand, the autocorrelation descriptors afforded the lowest performance with Ac, Sn, Sp and MCC of 78.48 ± 4.76%, 78.65 ± 5.66%, 78.89 ± 5.62% and 0.57±0.10, respectively. The predictive models built using CTD, Ctriad, QSO and PseAAC provided moderate performance with MCC of 0.60 ± 0.10, 0.62 ± 0.10, 0.63 ± 0.08 and 0.63 ± 0.09, respectively.

When looking into the relationship between protein sequence features and oligomerization, the phylogenetic relationships between sequences in the data sets should be taken into account. By not considering homologous relatedness amongst the FP samples, a problem in which FPs are the products of site-directed mutagenesis from a few wild-type sequences may arise. On the other hand, one site mutation may convert oligomeric FP to the monomeric state. For instance, the Ala206Lys mutation could convert a weakly oligomeric GFP to the monomeric form. Therefore, homologous reduction with the threshold of 100, 99 and 95% were considered.

Table 2 shows the results of the predictive models for the data set that removes all identical homologous sequence via the use of an identity threshold of 100% (non-redundant data set), it can be seen that the top performing tenfold CV set was built using amino acid composition, which afforded the highest performance with Ac, Sn, Sp and MCC of 83.07 ± 2.04%, 83.26 ± 2.19%, 82.95 ± 2.27% and 0.66 ± 0.04, respectively, whereas the performance of tenfold CV of autocorrelation descriptors (i.e. normalized Moreau-Broto autocorrelation, Moran autocorrelation and Geary autocorrelation) was 78.49 ± 4.76%, 78.36 ± 2.36%, 78.67 ± 2.30% and 0.57 ± 0.04 for Ac, Sn, Sp and MCC, respectively. However, the J48 model built with CTD, Conjoint, QSO and PseAAC were comparable with Ac, Sn, Sp and MCC in the ranges of 80.15–81.13, 79.66–81.19, 79.82–81.14 and 0.60–0.62, respectively.

**Table 3:** Summary of performance of QSAR models for predicting the oligomeric state of FPs (99% homologous sequence reduction) using the J48 algorithm

Descriptors	Training set				Tenfold CV set				External set			
	Ac (%)	Sn (%)	Sp (%)	MCC	Ac (%)	Sn (%)	Sp (%)	MCC	Ac (%)	Sn (%)	Sp (%)	MCC
AAC/DPC/TPC	98.22 ± 0.71	98.73 ± 0.89	97.69 ± 1.20	0.97 ± 0.01	79.40 ± 2.75	80.78 ± 1.86	77.98 ± 3.20	0.59 ± 0.06	80.78 ± 5.72	82.12 ± 6.28	80.12 ± 7.50	0.62 ± 0.12
AC	98.22 ± 0.71	98.73 ± 0.89	97.69 ± 1.20	0.96 ± 0.01	72.88 ± 3.46	74.52 ± 3.65	71.20 ± 3.74	0.46 ± 0.07	72.73 ± 6.11	74.89 ± 6.72	71.43 ± 7.57	0.46 ± 0.12
CTD	97.66 ± 0.90	98.06 ± 1.17	97.29 ± 1.46	0.95 ± 0.02	74.40 ± 3.03	75.01 ± 3.32	73.92 ± 3.42	0.49 ± 0.06	74.89 ± 5.79	75.76 ± 6.64	74.83 ± 6.95	0.50 ± 0.12
Ctriad	95.25 ± 1.87	96.62 ± 1.57	94.00 ± 3.64	0.91 ± 0.04	73.66 ± 2.84	75.78 ± 3.08	71.54 ± 3.15	0.47 ± 0.06	74.22 ± 6.02	77.26 ± 7.06	72.19 ± 7.21	0.49 ± 0.12
QSO	98.50 ± 0.64	98.63 ± 1.00	98.39 ± 1.15	0.97 ± 0.01	75.78 ± 2.71	77.37 ± 3.08	74.11 ± 2.74	0.51 ± 0.05	76.71 ± 5.27	78.92 ± 6.19	75.19 ± 6.35	0.54 ± 0.11
PseAAC	98.17 ± 0.74	98.38 ± 1.08	97.98 ± 1.36	0.96 ± 0.01	74.35 ± 3.00	75.87 ± 2.83	72.81 ± 3.67	0.49 ± 0.06	74.71 ± 5.74	77.17 ± 6.60	72.97 ± 6.93	0.50 ± 0.12

The performance of models with homologous sequence reduction set at 99% is shown in Table 3. Again, J48 model built using AAC/DPC/TPC descriptors was the top performing model as assessed via tenfold CV with Ac, Sn, Sp and MCC with 79.40 ± 2.75%, 80.78 ± 1.86%, 77.98 ± 3.20% and 0.59 ± 0.06, respectively, when compared to other J48 models built with different descriptor sets. As for the external set, the J48 model with the lowest performance was made with AC descriptors having Ac, Sn, Sp and MCC of 72.73 ± 6.11%, 74.89 ± 6.72%, 71.43 ± 7.57% and 0.46 ± 0.12, respectively. Nevertheless, it can be observed that J48 models built with different descriptor set performance well as assessed via tenfold CV set and external set.

**Table 4:** Summary of performance of QSAR models for predicting the oligomeric state of FPs (95% homologous sequence reduction) using the J48 algorithm

Descriptors	Training set				Tenfold CV set				External set			
	Ac (%)	Sn (%)	Sp (%)	MCC	Ac (%)	Sn (%)	Sp (%)	MCC	Ac (%)	Sn (%)	Sp (%)	MCC
AAC/DPC/TPC	97.54 ± 1.19	99.25 ± 0.90	94.85 ± 2.50	0.95 ± 0.03	72.13 ± 4.18	79.83 ± 3.66	61.03 ± 5.34	0.42 ± 0.09	72.89 ± 7.08	79.85 ± 6.92	64.16 ± 11.20	0.43 ± 0.15
AC	98.35 ± 0.87	99.31 ± 0.92	96.81 ± 1.95	0.97 ± 0.02	70.71 ± 4.45	77.73 ± 3.63	59.80 ± 6.10	0.38 ± 0.09	70.30 ± 8.55	77.40 ± 7.91	60.99 ± 13.19	0.38 ± 0.18
CTD	97.97 ± 1.06	98.33 ± 1.40	97.50 ± 1.95	0.96 ± 0.02	69.40 ± 4.95	75.24 ± 4.39	60.62 ± 6.33	0.39 ± 0.10	70.18 ± 7.79	75.54 ± 7.39	63.17 ± 12.39	0.38 ± 0.17
Ctriad	96.62 ± 1.33	98.07 ± 1.52	94.35 ± 2.89	0.93 ± 0.03	68.64 ± 5.99	76.28 ± 4.49	57.20 ± 8.12	0.34 ± 0.12	71.26 ± 8.36	78.04 ± 7.20	62.51 ± 12.24	0.40 ± 0.17
QSO	98.10 ± 1.08	98.55 ± 1.25	97.42 ± 2.33	0.96 ± 0.02	68.98 ± 4.21	76.15 ± 3.45	57.59 ± 5.63	0.34 ± 0.09	69.93 ± 6.90	77.19 ± 5.75	60.30 ± 11.15	0.37 ± 0.14
PseAAC	98.24 ± 0.92	98.38 ± 1.30	98.07 ± 1.71	0.96 ± 0.02	69.39 ± 4.97	76.34 ± 3.98	58.20 ± 6.67	0.35 ± 0.10	69.67 ± 8.03	76.92 ± 7.01	59.53 ± 10.36	0.36 ± 0.17

For the performance of the sequence homologous reduction at 95%, it can be seen that the top performing model of the tenfold CV set resulted in Ac, Sn, Sp and MCC of 72.13 ± 4.18%, 79.83 ± 3.66%, 61.03 ± 5.34% and 0.42 ± 0.09, respectively, was from the model built using amino acid composition. On the other hand, the other models built using different descriptors were comparable as shown in Table 4. As for the external set, again, model built with amino acid composition outperform others with Ac, Sn, Sp and MCC of 72.89 ± 7.08%, 79.85 ± 6.92%, 64.16 ± 11.20% and 0.43 ± 0.15, respectively.

### Identifying informative features

Investigating feature importance of each type of protein descriptor can provide insights into FP oligomerization. Herein, the efficient built-in feature importance selector of the DT algorithm was used. In the DT algorithm, the estimation of feature importance is calculated from the feature usage based on information gain. The feature with the highest usage score is the most important feature because it maximizes the prediction performance. Since amino acid composition provided the highest performance, it was selected as an input to explore important features for discriminating the oligomers from the monomers.

**Fig. 2:**
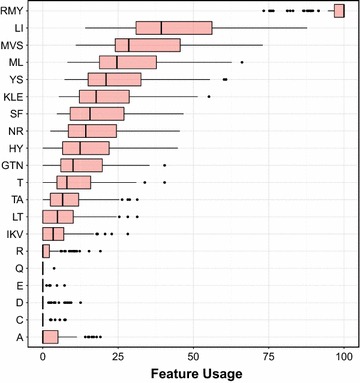
Box plot of the feature usage from the predictive model of FP oligomerization. Features with the highest usage is deemed to be the most important

Figure 2 demonstrates the top ten informative descriptors with the following feature usage: RMY ($95.96\pm 7.29$), LI ($44.56\pm 18.72$), MVS ($34.12\pm 15.28$), ML ($28.82\pm 14.14$), YS ($24.79\pm 12.90$), KLE ($21.38\pm 12.45$), SF ($19.28\pm 11.69$), NR ($17.01\pm 10.67$), HY ($14.92\pm 9.99$), GTN ($13.24\pm 9.58$) and T ($11.05\pm 3.34$). Notably, the top informative descriptors was the tripeptide RMY, which is comprised of the positively-charged Arg, the hydrophobic Met as well as the aromatic/hydrophobic Tyr. The second most important feature was the dipeptide LI, which are hydrophobic amino acids. Subsequent features from the top ten informative descriptors were also primarily hydrophobic in nature. This finding is corroborated by the experimental findings of Yarbrough et al. [33] in which the crystal structure of *Discosoma sp.* DsRed indicated that the oligomeric interfaces of subunits A and B consisted mostly of hydrophobic interaction along with a few hydrogen bonds and salt bridges. In a similar manner, the first discovered photoconvertible Kaede from *Trachyphyllia geoffroyi* displayed dominant hydrophobic interactions between the oligomeric interface at the A and C subunits [34]. Additionally, *Heteractis crispa* HcRed, the commercially available dimeric FP from Clontech, was converted to a dimer from a tetramer via the replacement of the hydrophobic Leu at position 123 to the aromatic His residue thereby perturbing the tetrameric hydrophobic interface. These findings reiterated that FP oligomerization are stabilized by several hydrophobic contact. Thus, hydrophobic residues at the interface were substituted with polar residues in attempt to create monomeric FPs [33–36]. Along with hydrophobic contacts, several other interactions including the formation of coordination bonds, ionic interactions, van der Waals’ contacts, electrostatic interactions, hydrogen bondings and $\pi$-$\pi$ stackings may mediate FP oligomerization at the oligomeric interface.

### osFP web server

To maximize the utility of the predictive model of FP oligomerization, a web server was developed using the Shiny package under the R programming environment. The utilization of Shiny boasts several benefits. The first advantage is the seamless integration of the web server with the aforementioned predictive model that was also built in R. The second benefit is that there is no requirement for developers to have an extensive knowledge of web development (i.e. although it may be useful). Thirdly, Shiny is platform-independent and can launch locally from any R environment (console R, RGui, RStudio, etc.) on any operating system whether Windows, Mac or Linux. Alternatively, users could also setup a remote server with installed instances of R and Shiny such that only a web browser is required to gain access to the application. As users can run their own instance of osFP, they can choose to customize the code to their own needs, run the application offline as well as ensuring strict privacy of the input data (i.e. it should be noted that the osFP web server does not cache or store the input data submitted by users). Most importantly, the fourth reason is that Shiny facilitates rapid development and deployment of web applications, which is especially beneficial for the scientific community as predictive models can be readily deployed as a web server, which is accessible to a wider group of users instead of confined to those with a background in computer science.

**Fig. 3:**
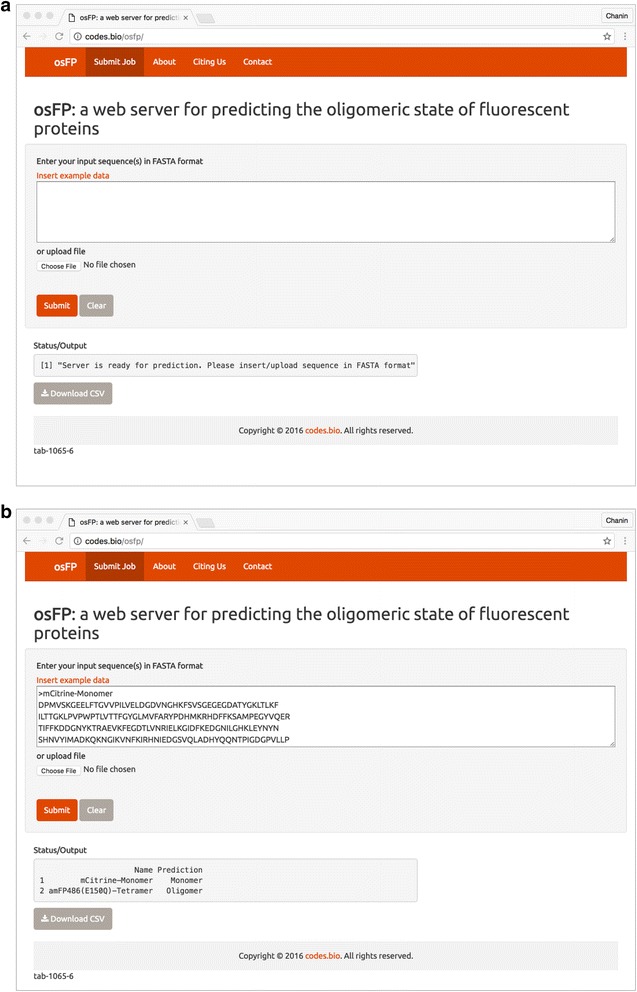
Screenshot of the osFP web server. Shown are the web server before (**a**) and after (**b**) prediction

The web server user interface accepts the input sequence data of FP in FASTA format and relays such information to the server script in which a predictive model is constructed and applied for classifying the input sequence(s) as being either monomeric or oligomeric. A screenshot of the osFP webserver is shown in Fig. 3. Under the hood, two R scripts are primarily responsible for driving the osFP web server along with the auxiliary role of the markdown files (e.g. about.md, cite.md and contact.md), which stores the content text that appears on the website. Firstly, the ui.R script performs as implied by its file name that is to house the user interface elements such as the website name, the navigation bar (i.e. links to the markdown file to display the respective constituent text appearing on the about, cite and contact tabs), the input text box, the file upload button, the *Insert example data* link, the submit button and the *Status/Output* text box. It should be noted that the website theme of the osFP web server is based on the shinythemes package in R, which at default makes use of the themes provided by Bootswatch (https://bootswatch.com/). These themes are written in Bootstrap (i.e. a HTML, CSS, and JS framework) that enable websites to be responsive and mobile-friendly (i.e. compresses the website width to fit onto a smart phone or tablet or expands the website width to fit the screen of a desktop or laptop monitor).

Secondly, the server.R script processes the data and builds the model as summarized by the following pseudocode:

1. Import R packages
2. Define function for computing amino acid based descriptors
3. Model building
  Accepts input FASTA sequence data from the text box or uploaded file
  Process the sequence data by computing the amino acid based descriptors for both the training and input data sets
  Combine descriptors and constructs the DT model using C4.5 algorithm
  Applies the constructed model to predict the oligomeric states of the input sequence data
4. Outputs the prediction results in an output text box on the webpage
5. Makes prediction results available for download as a CSV file.

The procedure for using the osFP web server is summarized below.

**Step 1.** Before starting the prediction, users should wait until the gray box that is found under the *Status/Output* heading shows the following text *Server is ready for prediction. Please insert/upload sequence in FASTA format*.

**Step 2.** Once the aforementioned message appears, users can enter their query sequence into the Input box or upload their sequence file by clicking on the *Choose file* button (i.e. found below the *Enter your input sequence(s) in FASTA format* heading). Finally, click on the *Submit* button to initiate the prediction process.

At the onset, users may also want to try out the functionality of the osFP web server via the use of an example input data by clicking on the *Insert example data* link. This calls upon the *updateTextInput* function from the Shiny package so as to insert the example FASTA data stored in the *fastaexample* variable into the input text box. Similarly, users can initiate the prediction process by clicking on the *Submit* button.

**Step 3.** The prediction results are automatically displayed in a gray box below the *Status/Output* heading. Users can also download the prediction results as a CSV file by clicking on the *Download CSV button*.

## Conclusion

This study represents the attempt in the development of a computational model for predicting and analyzing FP oligomerization from protein sequences using six classes of sequence descriptors consisting of AAC/DPC/TPC, AC, CTD, Ctriad, QSO and PseAAC. Findings indicated that the DT algorithm utilizing AAC/DPC/TPC (i.e. amino acid/peptide composition) outperformed the other descriptor class. Identification of informative features as obtained from the feature usage scores of DT revealed that the oligomeric interface are predominantly occupied by hydrophobic residues with a few electrostatic residues engaging in salt bridges. The results presented herein provide a glimpse on the important residues at the oligomeric interface that may be useful for guiding the rational design of monomeric forms of FP. To benefit the scientific community the predictive model was deployed as the osFP web server as well as providing the source codes and data sets on GitHub as to encourage further extension or adaptation of the web server. It is worthy to note that as new experimental data becomes available on the oligomeric states of FPs, the predictive model proposed herein could be continually updated by these growing data as to augment the model’s coverage and accuracy.
